# Evaluation of Low-Cost Phage-Based Microbial Source Tracking Tools for Elucidating Human Fecal Contamination Pathways in Kolkata, India

**DOI:** 10.3389/fmicb.2021.673604

**Published:** 2021-05-20

**Authors:** Renuka Kapoor, James Ebdon, Ashutosh Wadhwa, Goutam Chowdhury, Yuke Wang, Suraja J. Raj, Casey Siesel, Sarah E. Durry, Wolfgang Mairinger, Asish K. Mukhopadhyay, Suman Kanungo, Shanta Dutta, Christine L. Moe

**Affiliations:** ^1^Center for Global Safe Water, Sanitation and Hygiene, Rollins School of Public Health, Emory University, Atlanta, GA, United States; ^2^Environment and Public Health Research and Enterprise Group (EPHREG), University of Brighton, Brighton, United Kingdom; ^3^ICMR – National Institute of Cholera and Enteric Diseases (NICED), Kolkata, India

**Keywords:** fecal contamination, environmental surveillance, bacteriophages, GB-124, exposure pathways, transmission, low-income

## Abstract

Phages, such as those infecting *Bacteroides* spp., have been proven to be reliable indicators of human fecal contamination in microbial source tracking (MST) studies, and the efficacy of these MST markers found to vary geographically. This study reports the application and evaluation of candidate MST methods (phages infecting previously isolated *B. fragilis* strain GB-124, newly isolated *Bacteroides* strains (K10, K29, and K33) and recently isolated *Kluyvera intermedia* strain ASH-08), along with non-source specific somatic coliphages (SOMCPH infecting strain WG-5) and indicator bacteria (*Escherichia coli*) for identifying fecal contamination pathways in Kolkata, India. Source specificity of the phage-based methods was first tested using 60 known non-human fecal samples from common animals, before being evaluated with 56 known human samples (municipal sewage) collected during both the rainy and dry season. SOMCPH were present in 40-90% of samples from different animal species and in 100% of sewage samples. Phages infecting *Bacteroides* strain GB-124 were not detected from the majority (95%) of animal samples (except in three porcine samples) and were present in 93 and 71% of the sewage samples in the rainy and dry season (Mean = 1.42 and 1.83 log_10_PFU/100mL, respectively), though at lower levels than SOMCPH (Mean = 3.27 and 3.02 log_10_PFU/100mL, respectively). Phages infecting strain ASH-08 were detected in 89 and 96% of the sewage samples in the rainy and dry season, respectively, but were also present in all animal samples tested (except goats). Strains K10, K29, and K30 were not found to be useful MST markers due to low levels of phages and/or co-presence in non-human sources. GB-124 and SOMCPH were subsequently deployed within two low-income neighborhoods to determine the levels and origin of fecal contamination in 110 environmental samples. *E. coli*, SOMCPH, and phages of GB-124 were detected in 68, 42, and 28% of the samples, respectively. Analyses of 166 wastewater samples from shared community toilets and 21 samples from sewage pumping stations from the same districts showed that SOMCPH were present in 100% and GB-124 phages in 31% of shared toilet samples (Median = 5.59 and <1 log_10_ PFU/100 mL, respectively), and both SOMCPH and GB-124 phages were detected in 95% of pumping station samples (Median = 5.82 and 4.04 log_10_ PFU/100 mL, respectively). Our findings suggest that GB-124 and SOMCPH have utility as low-cost fecal indicator tools which can facilitate environmental surveillance of enteric organisms, elucidate human and non-human fecal exposure pathways, and inform interventions to mitigate exposure to fecal contamination in the residential environment of Kolkata, India.

## Graphical Abstract

Phages infecting *Bacteroides fragilis* strain GB-124 and non-source specific somatic coliphages (SOMCPH) were deployed for identification of fecal contamination pathways in Kolkata, India. Analyses of environmental samples representative of nine different exposure pathways, pooled sewage from shared community toilets and sewage from pumping stations showed the presence of both SOMCPH and GB-124 phages in all the sample types.

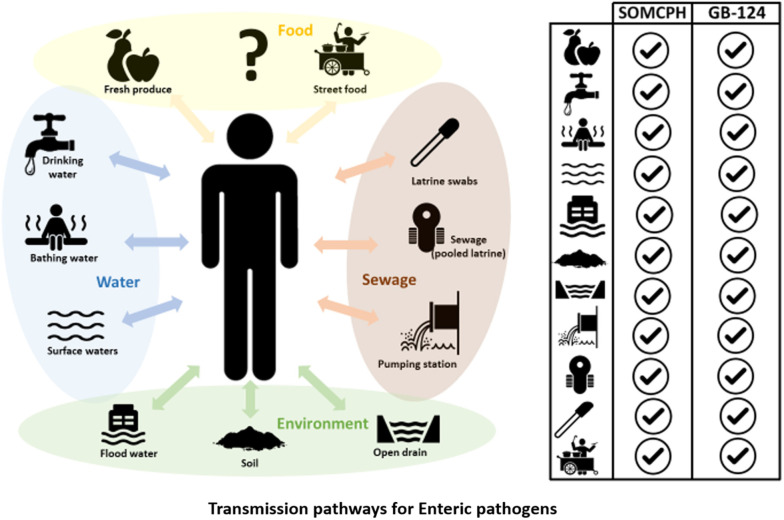

## Introduction

Exposure to enteric pathogens is associated with a significant health burden, largely borne by young children living in low-income settings (Kosek et al., 2003). Globally, enteric infections represent the third leading cause of death among children under five, accounting for approximately 589,000 deaths in 2017 (Global Burden of Disease Collaborative Network, 2018). Advances in methods to characterize human exposure to enteric pathogens have lagged behind, partly due to non-availability of resources in the settings where enteric diseases carry a disproportionally high burden of disease (Goddard et al., 2020).

In order to design prevention and control strategies necessary to reduce exposure to fecal contamination and enteric pathogens, there is a need to improve enteric disease surveillance strategies and understanding of the role of environment in the transmission of enteric pathogens. While clinical surveillance can be used to monitor the transmission of infection within a community, effective implementation of prevention and control strategies requires understanding the environmental transmission pathways for these pathogens. In addition to food and drinking water, soil, surface water, open drains, and sewage-irrigated raw produce can serve as vehicles of exposure to enteric pathogens (Wang et al., 2017; Bauza et al., 2020; Holcomb et al., 2020). However, all of these vehicles can become contaminated with fecal matter from a variety of sources (Bauza et al., 2020). This is especially true in high density, low-resource settings in low- and middle-income countries, where poor sanitation infrastructure and fecal sludge management, coupled with unsuitable urban animal husbandry practices and stray animal populations, contribute to fecal contamination of the environment by both human and non-human sources.

The majority of enteric disease surveillance programs focus on clinical surveillance of diarrheal cases. However, in the recent years, there has been an increasing paradigm shift towards improving environmental surveillance and running it in parallel with clinical surveillance (Asghar et al., 2014; Gumede et al., 2018; Andrews et al., 2020; Bivins et al., 2020; Matrajt et al., 2020). Samples from wastewater collection systems and treatment plants offer a means to effectively assess pathogen burden and transmission at a population level (Gardy and Loman, 2018).

Historically, fecal indicator bacteria (FIB), such as *E. coli* and enterococci have been used to evaluate both the sanitary quality of water and the performance of wastewater and drinking water treatment processes (World Health Organization (WHO), 2017). However, whilst FIB have been shown to exhibit broadly similar levels of inactivation by treatment processes and environment stresses as some bacterial pathogens, they are less indicative of more resistant microorganisms (e.g., viruses or protozoa). This has called into question their value as universal surrogate indicators for enteric pathogens (AWPRC Study Group on Health-Related Water Microbiology, 1991; Grabow et al., 2001; Jiang et al., 2001; McQuaig et al., 2006; Field and Samadpour, 2007; Stoeckel and Harwood, 2007; World Health Organization (WHO), 2017). Moreover, as FIB are widely distributed in both human and animal feces, they do not distinguish the source of contamination (Malakoff, 2002) and have even been shown to multiply in certain environmental niches (Solo-Gabriele et al., 2000; Vanden Heuvel et al., 2010; Sadowsky and Whitman, 2011). Therefore, FIB may not correlate well with the magnitude of human enteric pathogens in environmental samples, particularly when non-human fecal sources are present (Odagiri et al., 2016).

Despite recent advances in molecular-based diagnostic techniques for rapid genomic surveillance of specific enteric pathogens (Diemert and Yan, 2019; Saha et al., 2019) and more recently SARS-CoV-2 (Ahmed et al., 2020; Medema et al., 2020; Wu et al., 2020), technological difficulties associated with low or highly variable concentrations in samples (compared to gut commensal microbes), continues to make routine pathogen detection challenging and potentially costly, even in high-income countries. This is largely due to the need for concentration (composite sampling), enrichment, and selection steps prior to identification, combined with the fact that many infective agents display seasonal and/or spatial variations in their prevalence and individual variation in shedding rates (Sinclair et al., 2008; Gerba et al., 2017). Therefore, although pathogens can be detected using molecular methods, challenges relating to the complexity of sample matrices (e.g., water, sludge, sediments), representative sampling, and technological complexity (e.g., DNA/RNA extraction, processing, sequencing and analytical protocols), makes their deployment in many low-resource settings highly infeasible for practical and routine purposes (Raes and Bork, 2008; Kyle Bibby and Peccia, 2013), as such settings often lack basic laboratory capacity even for indicator monitoring or pathogen surveillance (e.g., lack of cold chain, electrical power, reliable reagent supplies etc.).

Limitations associated with existing FIB, combined with technical challenges and costs associated with the detection of specific pathogens has driven the development of microbial source tracking (MST) methods to distinguish sources of fecal contamination and understand possible exposure to pathogens in the environment and through food and water. This includes a range of non-molecular approaches, such as those involving the detection of specific bacteriophages (Puig et al., 1999; Payan et al., 2005; Ebdon et al., 2007, 2012; Wicki et al., 2011; Jofre et al., 2014; McMinn et al., 2014; Diston and Wicki, 2015; Dias et al., 2018) and molecular-based markers (e.g., Bacteroidales and crAssphage qPCR markers) which have shown considerable promise as MST markers (Reischer et al., 2013; Stachler et al., 2017; Ahmed et al., 2018a,b; Mayer et al., 2018). However, despite the fact that studies of pollution sources have been conducted in a range of rural and urban low-income settings (e.g., Kenya, India, Mozambique, Bangladesh, Chile) most MST studies have involved shipping samples to laboratories in high-income countries for analysis (Jenkins et al., 2009; Odagiri et al., 2015, 2016; Harris et al., 2016; Bauza et al., 2019; Fuhrmeister et al., 2019, 2020; Holcomb et al., 2020; Jennings et al., 2020).

This study sought to develop MST indicators for environmental samples in Kolkata, India so that the presence, magnitude, and origin of fecal contamination could be rapidly identified (within 18-24 h) by staff at the National Institute of Cholera and Enteric Diseases (NICED). This involved determining the potential suitability of non-molecular MST tools based on the detection of phages capable of infecting certain bacteria such as *Bacteroides* spp. Although the host-range of *Bacteroides* is not restricted to humans, phages infecting certain strains of *B. fragilis and B. thetaiotaomicron*, such as HB-13, HSP-40, GA-17, have been reported to be found almost exclusively in fecal material of human origin (Payan et al., 2005; Jofre et al., 2014). However, research has also shown that phages that are capable of lysing a specific host strain of *Bacteroides* in one part of the world, are not necessarily detected in samples of similar origin (or at similar levels) in other parts of the world (Kator and Rhodes, 1992; Chung et al., 1998; Puig et al., 1999; Payan et al., 2005; Blanch et al., 2006), suggesting that different host strains may be needed for meaningful MST studies in different catchments or geographic regions. However, phages infecting certain *B. fragilis* strains (e.g., GB-124) have been shown to be restricted to human hosts (Ogilvie et al., 2012, 2018) and have been previously reported in Europe and North and South America as a potentially low-cost human fecal indicator (Payan et al., 2005; Ebdon et al., 2007, 2012; McMinn et al., 2014, 2017; Prado et al., 2018). Geographic variations in the efficacy of MST markers have been reported in the extant literature (Ebdon and Taylor, 2006; Harwood et al., 2013) due to potential differences in host genetics, antibiotic usage, immunological factors and dietary differences. Consequently, it is essential to first validate the suitability of any MST approach in a given region, prior to deployment.

Here we report the development, evaluation, and suitability of low-cost candidate phage-based MST methods and non-source specific indicators for identifying fecal exposure pathways and sources in low-income urban districts of Kolkata. This study formed part of a larger SaniPath Typhoid project (grant number OPP1150697). The objectives of this study were to: (i) determine the ability of a well-established *Bacteroides* host strain (*B. fragilis* GB-124), less well-established *Kluyvera* host strain (*K. intermedia* ASH-08 - which had recently displayed promising human specificity in South East England) and newly-isolated *Bacteroides* hosts (K-10, K-29, and K-33) to sensitively and specifically quantify human fecal contamination through culture-based detection of phages infecting these strains in a range of environmental samples in Kolkata, India; (ii) deploy the most promising human-specific MST tool(s) and non-source specific indicators to identify fecal contamination sources in drinking water, bathing water, flood water, drain water, surface water, soil, raw produce, street food, and on swabs from shared community toilets in two low-income urban districts; and (iii) explore the influence of precipitation/season on the presence and levels of these phages in samples of municipal and domestic sewage and impacted surface waters from across the city.

## Materials and Methods

### Study Setting

Kolkata, capital of the State of West Bengal in India, is divided into 15 boroughs and 141 wards (administrative units) under the Kolkata Municipal Corporation (KMC) area. It is estimated that 32.4% of the total population of Kolkata lives in 5,500 overcrowded low-income areas (Ray, 2017) where inhabitants do not have access to basic amenities including improved water and sanitation. The study area included low-income neighborhoods within wards 58 and 59 in the eastern part of KMC; these areas are representative of other low-income parts of the city and are characterized by poor housing conditions, intermittent supply of piped municipal water, and multiple households sharing pour-flush toilets and water taps. Surface water bodies, such as canals and ponds, are commonly used for bathing and domestic hygiene. Livestock ownership (74% households, unpublished findings) is chiefly comprised of poultry, cattle, goats and pigs. Most household wastewater is collected by an underground piped sewerage system, and some is collected in open drains that overflow during the rainy season. Underground potable water distribution lines and sewerage pipes often lie close to each other and are prone to leakage and cross-contamination (Singh et al., 2015). Other factors known to facilitate the transmission of diarrheal pathogens, such as poor hygiene, shared sanitation facilities, and the lack of adequate hand washing/facilities are prevalent. Vending of prepared street food and fresh produce (cut fruit), is widely practiced, and young children and adults reported frequent consumption of these foods.

### Sample Collection

A total of 413 samples were collected and tested from a variety of known and unknown sources (animal feces, sewage, municipal wastewater, exposure pathways) over a 36-month period from July 2017 to March 2020.

#### Sewage Samples

In an attempt to isolate new *Bacteroides* host strains potentially suitable for phage detection in India and to establish whether the existing *B. fragilis* strain GB-124 and more recently isolated *K. intermedia* strain ASH-08 (which was isolated from raw municipal United Kingdom sewage and which appears to be capable of recovering high levels of phages in samples impacted by human fecal contamination in in South East England)could be used in this study setting, 100 mL sewage effluent samples were collected in 1 L sterile polyethylene bottles from the open canals, underground sewer manholes and open drains in the two study areas. Sample sites that were known to receive human fecal inputs were selected based on expert opinions of the field staff from NICED. All samples were kept on ice and transported to the lab for further processing within 4 h. Unfiltered sewage samples (*n* = 4, collected in Aug 2017) (serially diluted) were used for the isolation of potential *Bacteroides* host strains, whereas sewage samples [*n* = 56, collected between July-Aug 2017 (rainy) and March-May 2018 (dry season)] filtered through 0.22 μm polyvinylidene difluoride (PVDF) (or alternatively PES) membrane syringe filters (Millipore, USA) were used for evaluating the presence of phages against *B. fragilis* (GB-124) and *K. intermedia* (ASH-08) and SOMCPH(WG-5).

As part of our environmental surveillance study, two types of sewage samples were also collected, 1) wastewater accessed from manholes adjacent to shared toilets (*n* = 166 collected between Sept 2019 – March 2020) used by ∼ 100-250 residents in the two study neighborhoods, and 2) wastewater from selected pumping stations (*n* = 21 collected between Nov 2019 – March 2020) located across the city and representing excreta from >100,000 inhabitants. All samples were kept on ice and transported to the lab for further processing within 4 h of collection for enumeration of fecal indicator bacteria (FIB) (*E. coli*) and phages as described in sections “Enumeration of FIB (*E. coli*)” and “Enumeration of Bacteriophages”.

#### Animal Fecal Samples

A total of 60 fecal samples, comprised of 10 samples each from five common types of animals in the study neighborhoods (cattle, chicken, goats, pigs, and dogs) and an additional 10 ‘mixed animal’ fecal samples (containing samples of each of the five species) were analyzed to check the host specificity of *B. fragilis* (GB-124) and *K. intermedia* (ASH-08). On each occasion, two grams (wet weight) of animal feces was diluted 1:10 (w:v) in sterile DI water, vortexed until visible clumps were dispersed, and centrifuged at 4,500 x g for 10 min. The supernatant was filtered using a 0.22 μm PVDF (or alternatively PES) membrane syringe filter and analyzed for the presence of phages in accordance with standardized methods (section “Isolation of new Bacteroides hosts” below).

#### Environmental Samples

A key informant interview (KII) was conducted in each of the wards with a community leader (i.e., local political leaders or NGO representatives) who had a good understanding about the WASH facilities, environmental characteristics, and how community members interacted with their environment. The information from the KIIs, as well as self-reported exposure behavior information collected from people in the study neighborhoods, was used to select environmental samples representative of identified exposure pathways (along which fecal contaminants may be transmitted). Potential fecal exposure pathways included: (1) municipal drinking water supplied by KMC and accessed through public taps (both direct supply and stored household water), (2) water from both municipal and non-municipal supplies that was stored and used for bathing, (3) surface water from community ponds, (4) open drain water carrying liquid and solid waste, including rainwater, flood water, sewage and water from household activities, (5) flood water that remained stagnant for at least one hour after rain, (6) soil from communal areas where people gathered and/or children played, (7) swabs from walls and door handles of shared community toilets, (8) fresh produce that was commonly consumed raw as salad or garnish including tomato, cucumber, coriander leaves, etc. (9) street food sold on the streets and commonly consumed by children and adults including *phuchka* (a round, hollow pastry filled with a mixture of flavored water, tamarind sauce, chickpea, potato and onion), *chow-mein* (stir-fried noodles with vegetables) and cut fruit (e.g., water melon and pineapple). Sample collection was performed according to protocols described in the SaniPath tool that has previously been used to assess risk of exposure to fecal contamination across multiple pathways in several cities (Raj et al., 2020). All samples were kept on ice and transported to the lab for further processing within 4 h of collection for enumeration of FIB and phages as described in sections “Enumeration of FIB (*E. coli*)” and “Enumeration of Bacteriophages.”

### Isolation of New Bacteroides Hosts

The procedure to isolate new *Bacteroides* spp. was performed as described in Payan et al., 2005 and Livingston et al., 1978. 100 μL of unfiltered sewage was serially diluted and plated onto *Bacteroides* bile esculin (BBE) agar (Livingston et al., 1978) and incubated at 37°C overnight in anaerobic jars where anaerobiosis was achieved using commercial anaerobic generators (Anaerogen, Oxoid, United Kingdom). Esculin-positive (black) colonies were picked and plated for pure culture on BBE agar, both under aerobic and anaerobic conditions. A Gram stain test of isolates that grew under strict anaerobic conditions only was conducted to ensure that only Gram-negative obligate anaerobic rods were selected for subsequent phage testing. All potential host strains were grown in the *Bacteroides* phage recovery medium (BPRM) broth and tested for their ability to recover phage from known human and non-human fecal samples.

### Enumeration of FIB (*E. coli*)

All the environmental samples were processed within 6 h of collection and analyzed for *E. coli* using membrane filtration and m-ColiBlue24 broth (Hach, CO., United States) according to the manufacturer’s instructions. Each sample was analyzed in duplicate without dilution (varying volumes) and/or after dilution (1 ml volume) in phosphate buffered saline (PBS). Drinking and bathing water were analyzed without dilution in 100 mL and 10 mL volumes. Surface water and flood water samples were tested without dilution (10 mL, 1 mL) and at 1:10 and 1:10^2^ dilutions and drain water at 1:10^3^, 1:10^4^, and 1:10^5^ dilutions. Soil samples were mixed (10g/20 mL) in PBST (PBS with 0.04% of Tween 80) and homogenized with a shaker (Innova 2,300 platform shaker, Eppendorf, Inc., United States) for 30 min. The homogenized samples were allowed to settle for another 30 min, and the soil suspension was tested without dilution (1 mL) and at 1:10 and 1:10^2^ dilutions in PBS. Swabs (2 per sampling site) from community toilets shared by multiple households were washed in PBST (2 x 10 mL for each swab) and homogenized with a vortexer (Corning vortex mixer, Fisher Scientific, United States) for 30 s, followed by incubation at room temperature for 5 min and mixed again for another 30 s. After mixing, 1- and 10-mL volumes of the swab eluates were analyzed by membrane filtration. Fresh produce samples were rinsed with 500 mL PBST in a 2 L Whirl-Pak^TM^ bag and incubated at 37°C for 10 min, after which the bag was shaken manually for 30 s and was then massaged gently to dislodge the surface contamination. After shaking for an additional 30 s, the produce was removed from the Whirl-Pak^TM^ bag, weighed, and the eluate was analyzed without dilution (1 mL) and at 1:10 dilution in PBS. Street food samples were homogenized in PBST (25 g/225 mL) for 1 min using a sterile BagMixer bag and BagMixer 400 CC (Interscience Laboratory Inc., Woburn, MA) at speed 4 with gap at-3 mm. The homogenate was analyzed without dilution (1 mL) and at 1:10 dilution in PBS.

After processing, all the samples were filtered through 47 mm, 0.45 μm membrane filter (Millipore, United States) using a vacuum manifold (Tarsons, Kolkata, India). The filter was then placed in a 50 mm Petri dish containing a filter pad soaked with 2 ml of m-ColiBlue broth (Hach, CO., United States). The plates were incubated facing up at 37°C and examined after 24 h to record number of blue colonies (*E. coli*), reported as CFU (colony forming units). All water samples were reported as CFU/100 mL, soil and street food were reported as CFU/gram, fresh produce were reported as CFU/single serving, and shared household toilet swabs were reported as CFU/swab. Colony counts in the range of 1-200 were considered valid, and counts >200 were recorded as TNTC (too numerous to count). Plates showing smudged growth obscured by sample sediment were recorded as TDTC (too difficult to count), and plates with no growth on the membrane were recorded as ND (non-detectable).

### Enumeration of Bacteriophages

Samples were tested for phages infecting the newly isolated presumptive *Bacteroides* hosts, *B. fragilis* (GB-124), and SOMCPH (WG-5) using the double-agar-layer method as described in ISO-10705-4 (Anon, 2001) and ISO-10705-2 (Anon, 2000), respectively. *K. intermedia* (ASH-08) was cultured in accordance with ISO-10705-4 (Anon, 2001) and phages enumerated using the double-agar-layer method.

#### Preparation of Host Strain Cultures

Tubes with approximately 15 mL of BPRM broth (BPRMB) were inoculated with 1 mL of overnight culture of strains GB-124 and ASH-08. The tubes were filled completely with broth to ensure anaerobic conditions and incubated at 37°C until the culture reached an optical density of 0.33 at 620 nm. Similarly, tubes containing 10 mL modified Scholtens’ (MS) broth were inoculated with 1 mL of strain WG-5 (SOMCPH) and incubated at 37°C for growth to optical density of 0.33 at 600 nm. The cultures were placed on ice and used within 4 h.

#### Processing of Sewage and Environmental Samples

The sewage and aqueous environmental samples (drinking water, bathing water, surface water, open drain water, and flood water) were tested without any prior processing or dilution. Samples of fresh produce, street food and swabs from shared community toilets, were processed as described in section “Enumeration of FIB (*E. coli*)” and tested without dilution. For soil samples, 50 grams of soil was mixed with 100 mL of 10% beef extract (pH 7.2) and homogenized with a shaker (Innova 2,300 platform shaker, Eppendorf, Inc., United States) for 30 min. The homogenized samples were centrifuged at 1,500 *g* for 15 min, and the supernatant was filter sterilized (using 0.22 μm PVDF (or PES) membrane syringe filter).

Each sample was analyzed in duplicate (at either 1 mL and 100 μL volumes) and added to 1 mL of the host bacterium in a sterile 10 mL disposable test tube containing 2.5 mL of semi-solid BPRM agar (BPRMA) (for phages against GB-124 and ASH-08) or MS agar (MSA) (for SOMCPH). The contents of the tubes were gently vortexed and poured onto the surface of BPRMA plates supplemented with kanamycin monosulfate (100μg/mL) and MSA plates supplemented with nalidixic acid (100μg/mL). In order to increase the sensitivity of the assay in samples exhibiting low concentrations of phages, an increased sample volume (5 mL) was added to 5 mL of the *Bacteroides* and *Kluyvera* host strains and 7 mL of full strength BPRMA which was then poured onto a monolayer of BPRMA in a 90 mm Petri plate. All plates were left to set at room temperature, inverted and incubated at 37°C for 18-24 h either aerobically (MSA) or anaerobically (BPRMA) using anaerobic jars and anaerobic generators (Anaerogen, Oxoid, United Kingdom). Visible plaques (zones of lysis) in a confluent lawn of the host bacterium were counted as plaque forming units (PFU). PFU counts <300 were considered countable, counts >300 were recorded as TNTC and complete clearing of the host bacterial lawn was recorded as “complete lysis.”

### Phage Pre-enrichment

The presence/absence of phages infecting *B. fragilis* (GB-124) and SOMCPH in certain environmental samples where levels can be low (e.g., drinking water or street food), was also determined following a pre-enrichment step. However, whilst pre-enrichment is useful for detecting the presence/absence of a human signal in such samples where levels can be low, the presence/absence of SOMCPH is less useful compared to quantitative results for this general fecal indicator. Therefore, wherever possible it is better to use the quantitative, non-enriched counts for SOMCPH. Pre-enrichment was not used on environmental samples such as sewage from shared toilets and from pumping stations.

For pre-enrichment a 50 mL volume of the processed environmental sample was added to a 125 mL screw-capped Schott bottle (Millipore Sigma, United States) containing 60 mL double strength (ds) BPRMB and 15 mL of GB-124 culture (grown to optical density of 0.33 at 620 nm as described in section “Enumeration of Bacteriophages”). The bottle was filled completely with dsBPRMB to ensure anaerobic conditions, capped tightly, and incubated at 37°C for 18-21 h. Following overnight incubation, 5 mL of the enrichment culture was filtered using the 0.22 μm PVDF (or PES) membrane syringe filter into a centrifuge tube and centrifuged at 3,000 *g* for 5 min. 1 mL and 100 μL of the supernatant from the centrifuge tube was carefully added to 1 mL the host bacterium in a sterile 10 mL disposable test tubes containing 2.5 mL of semi-solid BPRMA (for GB-124) or MSA (for SOMCPH).

The contents of the test tubes were gently vortexed and poured onto the surface of BPRMA plates supplemented with kanamycin monosulfate (100 μg/mL) and MSA plates supplemented with nalidixic acid (100 μg/mL). The plates were left to set, inverted and incubated at 37°C for 18-24 h anaerobically (BPRMA) using commercial anaerobic jars and generators (Anaerogen, Oxoid, United Kingdom) or aerobically (MSA). The presence of phages resulted in the production of visible plaques (zones of lysis) in a confluent lawn of the host bacterium and the results were expressed as positive or negative for presence of plaques.

### Quality Control

Quality control measures were employed during both sample collection and lab analyses. One field blank of DI water was collected and processed on each sample collection trip. The sample collectors filled one 100 mL Whirl-Pak^TM^ bag with sterile distilled water at the sample collection site to assess their aseptic technique. This blank was tested in the laboratory for *E*. *coli*, and presence of any growth was considered as an indication of cross-contamination during sample collection process. One laboratory blank (Phosphate Buffered Saline) per laboratory technician per day, one negative control (sterile DI water) per batch of m-coliBlue, and one positive control for phage testing (B124-21, a purified *Siphoviridae* phage specifically infecting *Bacteroides* GB-124 strain) per day were processed for quality control. About 6.6% (28/426) of the field blanks were positive for *E. coli*, and these samples were removed from subsequent analysis. However, successful positive and negative phage controls (100% compliance) indicated that the NICED laboratory procedures met the study QA/QC criteria.

### Data Analysis

A value of 300 PFU was assigned to samples recorded as TNTC or “complete lysis” in phage enumeration assays. A value of 0.5 CFU was assigned for samples recorded as non-detectable and 200 CFU for samples recorded as TNTC or TDTC in the *E. coli* enumeration assay. The *E. coli* concentration was calculated using the recorded CFU and corresponding dilution factors. The following criteria was used for calculating the *E. coli* test results: (i) if the *E*. *coli* counts of all three dilutions were *<*1 CFU, the lowest diluted sample was used to estimate the concentration, (ii) if the *E*. *coli* counts of all three dilutions were *>*200 CFU, the highest dilution was used to estimate the concentration, (iii) if at least one *E*. *coli* count was within the detection limit (from 1 to 200 CFU) the average concentration of *E*. *coli* was calculated, ignoring the censored (out of detection limit) *E*. *coli* counts. Phage and *E. coli* concentrations for water and sewage samples were normalized to 100 mL and all concentrations were log_10_-tranformed before analyses. The Wilcoxon rank sum test was used to compare the differences in concentration of the three phage types by season, and the Spearman-rank test was used to examine correlation between the concentrations of phages (SOMCPH, phages of GB-124 and phages of ASH-08) in sewage samples. Logistic regression was used to test the association between *E. coli* concentration and presence/absence of SOMCPH and phages of *Bacteroides* strain GB-124 in different sample types. Results were considered significant at *p*-value <0.05. All statistical analyses were conducted using R software version 4.0.2 (R Core Team, 2020).

### Ethics

The study was reviewed and approved by the institutional review board at Emory University and the ethics committee at NICED. Written informed consent was obtained from the respondents at the time of surveys.

## Results

### Isolation of New Bacteroides Hosts

Processing and culture of four sewage samples from the city of Kolkata yielded three potentially useful anaerobic Gram-negative hosts (K-10, K-29 and K-33) from 33 presumptive *Bacteroides* isolates (Supplementary Information Table 1). However, further analysis revealed that isolates K-10, K-29, and K-33 were not capable of consistently recovering phages in 28 sewage samples (of known human origin) at concentrations similar to, or greater than strain GB-124 and so were not used beyond this point.

### Analyses of Fecal Samples From Animal Sources

To determine the host specificity of GB-124 and ASH-08, a total of 60 animal fecal samples were analyzed for phages against GB-124 and ASH-08 along with SOMCPH. SOMCPH were detected in a majority (40 to 90%) of animal fecal samples at concentrations ranging from 3.47-7.02 log_10_ PFU/gm of feces, while phages against ASH-08 were detected in 20 to 80% of animal samples (except goat) at concentrations ranging from 2.30-7.21 log_10_ PFU/gm of feces. Phages infecting GB-124 strain were not detected in any of the animal fecal samples, except for three pig feces samples (Mean = 3.64 log_10_ PFU/gm of feces) (Table 1).

**TABLE 1:** Analyses of samples from animal and human origin for phages against *B. fragilis, K. intermedia*, and somatic coliphages (SOMCPH) in Kolkata (*n* = 116).

**Source**	**N**	***Bacteroides* phages (strain GB-124)**			***K. intermedia* phages (strain ASH-08)**			**SOMCPH (strain WG-5)**		
		**Log_10_ PFU per gram of feces**		**% of positive samples**	**Log_10_ PFU per gram of feces**		**% of positive samples**	**Log_10_ PFU per gram of feces**		**% of positive samples**
		**Mean**	**Range**		**Mean**	**Range**		**Mean**	**Range**	
Cattle	10	ND	ND	0	3.95	2.30-4.34	40	4.14	2.70-4.83	60
Goat	10	ND	ND	0	ND	ND	0	3.47	2.60-3.78	40
Pig	10	3.64	3.15-4.32	30	6.38	3.70-7.21	80	6.41	5.48-7.00	70
Dog	10	ND	ND	0	4.54	3.00-4.83	20	7.02	2.00-7.67	90
Chicken	10	ND	ND	0	5.63	5.21-5.95	50	6.20	3.23-7.04	90
Pooled animal*	10	3.95	−	10	6.08	5.60-6.43	100	6.58	5.91-6.86	100
Human (Sewage)	56	1.59	0.60-3.60	79	3.00	1.60-5.15	93	3.15	1.08-5.58	100

PFU = Plaque Forming units; N, number of samples tested; ND, not detected (i.e., below detection threshold (<1 PFU/gram of feces). *fecal samples from the five animal species were mixed to generate pooled samples.

### Analyses of Sewage Samples From Known Human Sources

**FIGURE 1:**
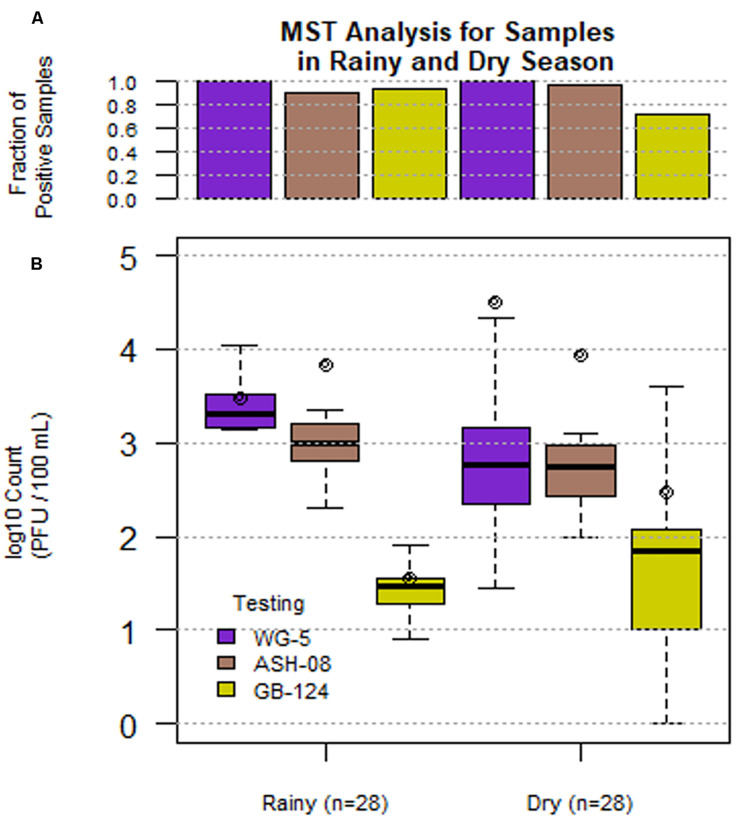
Phage (SOMCPH (WG-5), *K. intermedia* (ASH-08) and *B. fragilis* (GB-124)) presence **(A)** and concentrations **(B)** in sewage samples (*n* = 56) during rainy (July-Aug 2017) and dry season (March-May 2018) from two study neighborhoods in Kolkata.

A total of 56 sewage samples, 28 each during the rainy season (July-Aug 2017) and dry season (March to May 2018) were collected from the two wards in Kolkata. SOMCPH were detected in all samples, regardless of season (Table 1 and Figure 1A), whereas phages infecting ASH-08 were detected in 89% (25/28) and 96% (27/28) of samples in rainy and dry season, respectively. Similarly, phages against GB-124 were detected in 93% (26/28) and 71% (20/28) of the samples in rainy and dry season, respectively (Figure 1A).

In the sewage samples, SOMCPH concentrations [(mean (range)] were highest [3.15 log_10_ PFU/100 mL (1.08 to 5.58)], followed by phages infecting ASH-08 [3.00 log_10_ PFU/100 mL (1.60 to 5.15)] (Table 1). The overall concentrations of phages of GB-124 [1.59 log_10_ PFU/100 mL (0.60 to 3.6)] were significantly (*p* value < 0.001) lower than those of the non-source-specific phages (SOMCPH and phages against ASH-08). Further analyses showed a seasonal variation in the concentrations of all the three phage types (Figure 1B). Concentrations of SOMCPH were significantly higher (*p* value = 0.01) during rainy season [3.27 log_10_ PFU/100 mL (2.30 to 4.03)] compared to dry season [3.02 log_10_ PFU/100 mL (1.08 to 5.58)]. Concentration of phages infecting ASH-08 were also higher in the rainy season [3.15 log_10_ PFU/100 mL (2.30 to 4.70)] compared to the dry season [2.86 log_10_ PFU/100 mL (1.60 to 5.15)]. The concentrations of phages of GB-124 were consistently the lowest when compared to SOMCPH and ASH-08 phages, both during rainy [1.42 log_10_ PFU/100 mL (0.60 to 2.35) and dry season [1.83 log_10_ PFU/100 mL (0.60 to 3.60)]. There was no significant correlation between concentrations of the three phage groups.

### Analyses of Environmental and Sewage Samples

#### FIB (*E. coli*)

A total of 110 environmental samples (ranging from 2 to 22 samples per pathway) from the nine exposure pathways were collected over a period of 11 months (from May 2019 to March 2020). *E. coli* were detected in all samples of drain water and flood water, the majority (92%) of the swabs from shared community toilets, 79% of surface water, nearly 70% of soil, produce and street food samples, 27% of bathing water, and 17% of drinking water samples (Table 2). As expected, drain water had the highest level of *E*. *coli* [mean (SD)] [7.07 log_10_ CFU/100 mL (1.25)], followed by high concentrations in flood water [4.63 log_10_ CFU/100 mL (0.46)] and surface water [4.2 log_10_ CFU/100 mL (1.25)]. The samples of drinking and bathing water had the lowest frequency of *E. coli* detection and the lowest mean concentrations [0.92 log_10_ CFU/100 mL (1.5)] and [0.93 log_10_ CFU/100 mL (0.58), respectively]. Fresh produce displayed substantial fecal contamination as indicated by mean *E. coli* concentration of 3.7 log_10_ CFU/serving but street food was less contaminated [1.61 log_10_ CFU/gram (1.26)]. Soil samples from public areas where people gathered and/or children played were also highly contaminated with feces [mean *E. coli* 3.37 log_10_ CFU/gram]. However, swabs from surfaces likely to be touched in shared community toilets had only moderate *E. coli* levels [mean 1.78 log_10_ CFU/swab]. Of the 187 sewage samples collected as part of the SaniPath Typhoid environmental surveillance program over a period of 8 months (from August 2019 to March 2020), 95% of pumping station and 89% of the shared community toilet samples had similar high concentrations of *E. coli* [6.37 log_10_ CFU/100 mL (0.67) and 6.79 log_10_ CFU/100 mL (1.11), respectively] (Table 2).

**TABLE 2:** Fecal indicator bacteria (*E. coli*) and phages (SOMCPH and GB-124) detection (with and without enrichment) in environmental samples from two study neighborhoods and selected municipal pumping station samples in Kolkata (*n* = 297).

**Sample type**	**Total (N)**	**n/N (%) positive for *E. coli***		**n/N (%) positive for SOMCPH (WG-5)**		**n/N (%) positive for *Bacteroides* phage (GB-124)**	
		**n (%) positive**	**Log_10_ CFU/100 mL Mean (SD)**	**Without enrichment**	**With enrichment**	**Without enrichment**	**With enrichment**
Drinking water	11	3 (27)	0.92 (1.50)	1/11 (9)	1/8 (13)	0/11 (0)	1/8 (13)
Bathing water	6	1 (17)	0.93 (0.58)	1/6 (17)	NT	1/6 (17)	NT
Surface water	14	11 (79)	4.20 (1.25)	10/14 (71)	7/8 (87)	6/14 (43)	5/8 (62)
Open drain	6	6 (100)	7.07 (1.25)	4/6 (67)	4/6 (67)	2/6 (33)	3/6 (50)
Flood water	2	2 (100)	4.63 (0.46)	2/2 (100)	2/2 (100)	0/2 (0)	2/2 (100)
Produce	22	15 (68)	3.70 (1.80)	6/22 (27)	3/14 (21)	1/22 (5)	0/14 (0)
Street food	15	10 (67)	1.61 (1.26)	3/12 (25)	4/13 (31)	1/12 (8)	3/13 (23)
Soil	21	15 (71)	3.37 (1.12)	10/21 (48)	7/14 (50)	5/21 (24)	4/14 (29)
Swabs from shared toilets	13	12 (92)	1.78 (1.36)	0/10 (0)	1/4 (25)	0/10 (0)	1/4 (25)
Total	110	75 (68)		37/104 (36)	29/69 (42)	16/104 (15)	19/69 (28)
Sewage from shared toilets	166	148 (89)	6.79 (1.11)	166/166 (100)	NT	51/166 (31)	NT
Sewage from pumping station	21	20 (95)	6.37 (0.67)	20/21 (95)	NT	20/21 (95)	NT
Total	187	168 (90)		186/187 (99)		71/187 (38)	

*N, Total number; n, number positive; CFU, Colony forming units; SD, standard deviation. NT, not tested.*

#### Phages (SOMCPH and GB-124)

A total of 104 environmental samples from potential exposure pathways were tested for the presence of SOMCPH and phages infecting strain GB-124. Overall, SOMCPH were detected in 36% of these samples, while 15% tested positive for phages infecting GB-124 without pre-enrichment. The highest concentrations [(mean (range)] of SOMCPH and phages of GB-124 were detected in samples from open drain [5.29 log_10_ PFU/100 mL (4.74 to 5.48)] and [5.06 log_10_ PFU/100 mL (4.22 to 5.48), respectively] whilst the lowest concentrations of SOMCPH and phages of GB-124 were detected in samples of soil [1.96 log_10_ PFU/gram (0.30 to 3.29) and [1.67 log_10_ PFU/gram (0.40 to 3.78), respectively]. In order to increase the phage detection rates, a pre-enrichment step, followed by a concentration process (as described in section “Phage Pre-enrichment”) was applied to a set of 69 samples. These overall detection rates using this enhanced detection approach increased from 36 to 42% and 15 to 28% for SOMCPH and GB-124, respectively, and both phage groups were detected in samples from all potential fecal exposure pathways except for GB-124 phage in produce (Table 2).

A total of 187 sewage samples were analyzed without the enrichment step because we expected both SOMCPH and GB-124 phages to be present in high concentrations. All of the samples from shared community toilets were positive for SOMCPH, but only 31% tested positive for phages infecting strain GB-124 (Median = 5.59 log_10_ PFU/100 mL and < 1 log_10_ PFU/100 mL, respectively). Almost all (95%) of the pumping station samples tested positive for both SOMCPH and phages of GB-124 (Median = 5.82 log_10_ PFU/100 mL and 4.04 log_10_ PFU/100 mL, respectively) (Table 2).

### Association Between FIB and Phages

Logistic regression analyses indicated that there was no significant association between the concentration of *E. coli* and the odds of detecting SOMCPH in any of the environmental samples, except soil [Odds ratio (OR) (95% CI) = 3.32 (1.13, 9.75); *p*-value = 0.029]. Similarly, there was no significant association between *E. coli* concentrations and detection of phages of GB-124, with the exception of sewage from shared community toilets [OR (95% CI) = 1.17 (1.22, 2.36); *p*-value = 0.002] (Supplementary Information Table 2).

## Discussion

The overall goal of this study was to establish low-cost, geographically suitable tools to identify potential pathways of exposure to human fecal contamination that could serve as transmission routes of typhoid fever in Kolkata. Whilst phages against *Bacteroides* strain GB-124 had previously been successfully detected in municipal sewage (untreated and treated) from England, Cuba, Ireland, France, Portugal, Denmark, Brazil, Spain, Italy, United States, and Uganda (Ebdon et al., 2007, 2012; Vijayavel et al., 2010; McMinn et al., 2014; Dias et al., 2015, 2018; Purnell et al., 2016; Prado et al., 2018), the suitability of phages infecting GB-124 as indicators of human fecal contamination in India had not been characterized. The consistent detection of phages of GB-124 in sewage and a wide range of fecally contaminated environmental matrices, and its absence from pooled cattle, chicken, goat and dog feces suggest its potential as a culture-based indicator of human fecal contamination in Kolkata. Whilst this study did not yield new additional *Bacteroides* hosts suitable for recovering phages in Kolkata, the results of GB-124 deployment in this setting were promising for many types of environmental samples.

***Specificity:*** Phages infecting GB-124 host strain were detected in the majority of the environmental samples with known human fecal contamination sources (sewage samples) and they were absent from the majority of fecal samples from common animal species in this setting (with the exception of three porcine fecal samples), despite close co-habitation of humans and animals in the study neighborhoods. Our findings are consistent with other studies where GB-124 phages were either not detected or detected at very low levels (close to the limit of detection) in non-human samples (Payan et al., 2005; Ebdon et al., 2007, 2012; Ogilvie et al., 2012; Harwood et al., 2013; McMinn et al., 2014; Diston and Wicki, 2015). These findings are also in line with the observations of Jebri et al. (2017), who suggest that source specificity is not absolute and, though seldom, *Bacteroides* host strains detect very low numbers of phages in the non-corresponding sources. Another possible reason for the presence of the GB-124 phages in a few porcine fecal samples could be the result of cross-contamination of porcine and human feces at the sample collection sites which were located in densely-populated household compounds. Interestingly, phages against *K. intermedia* (ASH-08) were also detected in non-human sources in Kolkata (with the exception of goats), which was not the case when this host was originally developed by the authors in the United Kingdom (data not shown), indicating a lack of discriminatory power (source specificity) and unsuitability for MST in Kolkata. On the other hand, SOMCPH were consistently detected in both human and non-human fecally contaminated samples (e.g., sewage and animal feces) indicating its utility as indicator of general (non-source specific) fecal contamination.

***Sensitivity:*** Our analyses showed variable detection of phages in certain samples such as individual fecal samples (Table 1) as observed in other studies (Tartera and Jofre, 1987). This is not entirely unexpected since an individual fecal sample taken at a single point in time (during which phages may or may not be present), is unlikely to be representative of the total intestinal microbiota, or an accurate assessment of community carriage and shedding. This is not in detriment of the prevalence of the phages but suggests that pooled samples and sewage (mixtures of numerous individuals) are more consistently likely to contain phages and as such may be more reliable MST targets. Our analyses of sewage samples showed the presence of all the three phage types, with levels of SOMCPH and phages of GB-124 similar to those reported earlier from untreated sewage (Ebdon et al., 2012; Dias et al., 2015, 2018; Purnell et al., 2015, 2016; McMinn et al., 2017). As expected, phages of GB-124 were present at lower concentrations than SOMCPH in sewage (Payan et al., 2005; Blanch et al., 2006; Ebdon et al., 2007, 2012; Nnane et al., 2012; Harwood et al., 2013; McMinn et al., 2014; Diston and Wicki, 2015) and were consistent with concentrations reported from Europe and United States of 3.90 log_10_ PFU/100 mL and 2.58 log_10_ PFU/100mL, respectively. The SOMCPH concentrations we detected in sewage in Kolkata were also similar to the concentrations observed in Europe [approx. 5.95 log_10_PFU/100mL] and the US [approx. 3.71 log_10_PFU/100mL] (Ebdon et al., 2007, 2012; McMinn et al., 2014, 2017; Dias et al., 2015; Purnell et al., 2015, 2016). The higher concentrations of SOMCPH are partly due to the fact that they are indicators of general fecal contamination and are not restricted to only human hosts, but all warm-blooded animals. Lower *Bacteroides* concentrations in some environmental sample types, such as sewage impacted surface waters, may be associated with increased rates of bacterial die-off due to greater sensitivity to temperature, the presence of oxygen and grazing by predators in certain environmental matrices (Ballesté and Blanch, 2010). No statistically significant correlation between concentrations of the three phage groups (SOMCPH, ASH-08 and GB-124) was found during this study. These findings are consistent with those of McMinn et al. (2014), who did not detect any significant correlation between concentrations of phages of GB-124 and SOMCPH during year-long study of wastewater effluents across the US. The seasonal variation in phage concentrations observed in Kolkata was similar for SOMCPH, but for GB-124 phages, contrasted with the findings of Ebdon et al. (2007), who reported higher concentrations of both SOMCPH and GB-124 in river water during high rainfall events as compared to low rainfall events. In Kolkata, the concentration of human fecal contamination in sewage may become more dilute because of the continued input of large volumes of rainwater into the sewers and canals during monsoon period. Whereas in some other settings, and especially during the ‘first flush’ rainfall may wash additional fecal contamination from land run-off into surface waters.

Lower concentrations of phages of GB-124 and SOMCPH compared to *E. coli* observed in certain matrices drove the need for the pre-enrichment and concentration process. This method modification appeared to enhance overall detection rates of SOMCPH and phages infecting GB-124, particularly in some specific types of samples (e.g., drinking water, surface water, open drain, flood water, street food, soil, and swabs from shared community toilets for phages infecting GB-124; and drinking water, surface water, street food, soil and swabs from shared community toilets for SOMCPH) as compared to phage detection without enrichment. However, the small sample sizes for many of the different environmental matrices make it difficult to draw conclusions about the impact of this method modification for specific types of samples. The pre-enrichment step is therefore useful to determine presence or absence of MST markers (human signal) but might not be useful for SOMCPH when quantification of these indicators is required to ascertain the actual levels of fecal contamination in a given sample.

Detection of phages infecting GB-124 from municipal sewage samples collected at pumping stations was far greater (95%) than detection from wastewater samples collected from manholes adjacent to shared community toilets (31%), although the samples from the shared toilets were clearly from human fecal sources and spent less time in transit through sewage lines. This finding could possibly be due to the lower number of individuals contributing to the wastewater near the shared community toilets compared to municipal sewage from large catchment populations. Also, difficulty obtaining sufficient volumes of wastewater from the manholes meant that sample collectors sometimes added additional flush water to the nearest toilet, and this may have impacted the quality of the sample.

***Phage-based MST approaches***: Bacteriophages are attractive fecal indicators and MST tools for low-resource settings because they are relatively simple, low-cost, and do not require advanced laboratory conditions like PCR-based MST, or pathogen detection. Whilst SOMCPH have successfully been used as surrogates of pathogenic viruses with which to assess the efficacy of riverbank filtration of highly polluted surface waters in Delhi (Sprenger et al., 2014), this is the first study to describe the successful use of *Bacteroides* GB-124 phages for MST in India. Previous MST studies from India have used either antibiotic resistance analysis and DNA fingerprinting-based methods (Murugan et al., 2012), or *Bacteroidales* qPCR using human BacHum and animal markers (BacCow for ruminants, BacCan for canines) (Odagiri et al., 2015, 2016; Schriewer et al., 2015). For example, Odagiri et al. (2015, 2016) used molecular-based *Bacteroidales* fecal markers to measure human and domestic animal fecal contamination in community tube wells and ponds (*n* = 301) and to assess multiple exposure pathways in homes (*n* = 354), in rural settings in Odisha. However, whilst fecal indicator bacteria were detected and enumerated in country in this cross-sectional study of 60 villages in India, the quantitative PCR assays for the MST indicators (BacUni, BacHum and BacCow, BacCan) were performed on samples that were stored on ice for up to 2 months, prior to being sent to the United States for analysis. Similarly, molecular-based MST studies that have been conducted in a variety of rural and urban low-income settings, including Kenya, Mozambique, Bangladesh, and Chile, relied on shipping the samples to a lab in a high-income country to perform the analyses (Jenkins et al., 2009; Harris et al., 2016; Bauza et al., 2019; Fuhrmeister et al., 2019, 2020; Holcomb et al., 2020; Jennings et al., 2020). The findings of this Kolkata study are significant as they demonstrate that a phage-based MST assay offers a low-cost, rapid (18 h) MST approach that laboratories in the region can employ to elucidate human and non-human fecal contamination sources and transmission pathways in complex urban environments such as those present in Kolkata, India. Furthermore, phage-based MST methods such as those reported here are based on the detection of infectious viral particles within a sample (rather than fragments of DNA/RNA) and hence may provide additional information for making inferences about the potential viability and infectivity of other microorganisms (e.g., enteric pathogens) also present.

## Conclusion

This is the first published study on the use of *Bacteroides* host strain GB-124 as an indicator of human fecal contamination in India. GB-124-based MST was found to be suitable for identifying potential exposure to human fecal contamination in densely populated, low-resource urban settings. Culture-based detection of GB-124 phage, coupled with the pre-enrichment and concentration steps described in this study, can be used by trained staff to routinely detect human fecal contamination in environmental samples, and can serve as a low-cost screening tool to enable more efficient environmental surveillance for human enteric pathogens by identifying subsets of samples that should be targeted for more advanced analyses for the detection of human-specific pathogens such as *S*. Typhi. The low-cost phage-based tools (GB-124 and SOMCPH) demonstrated here have potential to support fecal exposure assessments and evaluations of community-based sanitation and fecal sludge management interventions.

## Data Availability Statement

Raw data was generated at the National Institute of Cholera and Enteric Diseases (NICED), Kolkata, India. Derived data supporting the findings of this study are available from the corresponding author (CM) upon reasonable request.

## Author Contributions

RK and JE conceived and designed the experiments and prepared the manuscript. JE, RK, AW, and GC conducted the validation studies and trained the lab team. SK facilitated the study site selection and supervised field activities and sample collection. AM supervised the lab analyses of the field samples. SR, CS, and SED trained the field team in sample collection. WM designed data collection forms and conducted data analyses. WM and CS performed data monitoring. YW conducted data analyses. CM and SD conceived of and supervised the overall study. All authors contributed to the article and approved the submitted version.

## Conflict of Interest

The authors declare that the research was conducted in the absence of any commercial or financial relationships that could be construed as a potential conflict of interest.
