# Supplementary material for: Evaluation of Low-Cost Phage-Based Microbial Source Tracking Tools for Elucidating Human Fecal Contamination Pathways in Kolkata, India
Source: Front Microbiol. 2021 May 20;12:673604. doi: 10.3389/fmicb.2021.673604 (PMC8173070; doi:10.3389/fmicb.2021.673604)
Supplement: Supplementary file 1 [file Table_1.pdf]

Table S1. Analyses of sewage samples for isolation of new *Bacteroides* host strain in Kolkata

**Origin**	**Number of Sewage Samples**[§]	**Number of isolates**	**Number of strict anaerobes**	**Number of Gram-negative facultative anaerobes (%)**	**Strain designation**
Area 1	1	4	0	0 (0)	-
Area 2	1	12	1	1 (8)	K-10
Area 3	2	17	2	2 (12)	K-29, K-33

§ included samples from open canal, underground sewers and open drains

Table S2. Association between fecal indicator bacteria (*E. coli*) concentration and phage (WG-5 and GB-124) detection in environmental samples from two study neighborhoods and selected municipal pumping stations

**Sample Type**	**[¶]Odds Ratio (95% CI) for WG-5**				**[¶]Odds Ratio (95% CI) for GB-124**			
	**[§]Without enrichment**	***p value**	**[§]With enrichment**	***p value**	**Without enrichment**	***p value**	**With enrichment**	***p value**
Drinking Water	UD	-	UD	-	UD	-	UD	-
Bathing Water	UD	-	NT	-	UD	-	NT	-
Surface Water	0.77 (0.16, 3.68)	0.740	UD	-	1 (0.26, 3.82)	0.995	UD	-
Open Drain	UD	-	UD	-	UD	-	UD	-
Flood water	UD	-	UD	-	UD	-	UD	-
Raw Produce	1.16 (0.61, 2.2)	0.652	4.14 (0.2, 83.85)	0.355	UD	-	UD	-
Street Food	2.41 (0.79, 7.34)	0.122	2.9 (0.77, 11)	0.117	0.32 (0.01, 14.27)	0.554	1.58 (0.52, 4.8)	0.421
Soil	3.32 (1.13, 9.75)	**0.029**	3.01 (0.94, 9.64)	0.064	2.94 (0.87, 9.86)	0.081	16.86 (0.17, 1652.74)	0.227
Shared toilets swabs	2.06 (0.61, 6.94)	0.242	UD	-	UD	-	UD	-
Shared toilets (sewage)	UD	-	NT	-	1.7 (1.22, 2.36)	**0.002**	NT	-
Pumping Station (sewage)	26.19 (0.11, 6048.33)	0.240	NT	-	28.14 (0.12, 6690.47)	0.232	NT	-

[¶] Odds ratio and Confidence intervals (CI) from logistic regression analyses

* results considered significant at p value < 0.05 are shown in **bold**

[§] UD, undetermined due to small sample size or because results for phage detection were all either positive or negative; NT, not tested
